# Application of rules 1 to 6 for maximizing government infrastructure utilization in laparoscopic cholecystectomy: A case series analysis

**DOI:** 10.6026/973206300213855

**Published:** 2025-10-31

**Authors:** Anil Kumar, Shagufta Naaz, Ravi Shekhar, Abhyuday Kumar, Shiv Kishor, Rekha Kumari

**Affiliations:** 1 Department of Trauma Surgery & Critical Care, All India Institute of Medical Sciences, Patna, India; 2 Department of General Anaesthesia, All India Institute of Medical Sciences, Patna, India; 3 Department of General Surgery, All India Institute of Medical Sciences, Patna, India

**Keywords:** Laparoscopic, cholecystectomy, LC, 1 to 6 rules, GSD, government hospital

## Abstract

Laparoscopic cholecystectomy (LC) is the gold standard treatment for gallstone disease, but long waiting lists are common in India due to high disease prevalence and limited government resources. Therefore, it is of interest to evaluate a two-operation room (OR) model to optimise resource utilisation, in which patient preparation in one OR was overlapped with surgical steps in another OR. A total of 64 patients were operated on over three days, with an average operative time of 24.45 minutes per case; significantly more surgeries were completed in 6 hours with the new model (21.3 cases) compared to routine practice (6.3 cases in 8 hours, p <0.05). Most patients were female (98%), with a mean age of 44.8 years and operative times ranged from 9 to 55 minutes. The "1-6 rule" (1 consultant, 2 ORs, 3 assistants, 4 anaesthetists, 5 nursing/support staff and 6 hours of surgery) may be an innovative policy strategy for maximising LC efficiency in government hospitals.

## Background:

Laparoscopic cholecystectomy (LC) is the accepted gold-standard treatment for gallstone disease (GSD) because it is minimally invasive and enables faster recovery compared with open surgery [1]. In India, gallstone disease is highly prevalent, particularly among women and contributes substantially to the surgical workload in public hospitals [2, 3]. Long waiting lists for LC are frequently observed in government medical colleges due to high case volumes, limited operating room capacity and competing surgical priorities [4]. In many centres, acute cholecystitis is managed conservatively and patients are scheduled for delayed interval LC rather than early surgery, further prolonging waiting times [5]. In addition, operating theatre utilization and team structure strongly influence throughput and efficiency in high-volume Indian hospitals [6]. Therefore, it is of interest to describe an innovative operating-room model that may maximize available resources and increase the number of LCs performed in government settings.

## Methods and Materials:

This observational study was carried out at All India Institute of Medical Sciences, Patna. The patient with diagnosis of GSD on the basis of clinical symptoms and ultrasound findings were included in the study. The patients who were less than 20 years and older than 80 years, pregnancy with GSD, gall bladder mass on ultrasound, acute pancreatitis and carcinoma of gall bladder were excluded from the study. All basic investigations including complete blood count, liver function test, kidney function test, blood sugar level, chest X- Ray, electrocardiography and viral markers were advised. In second visit, when all laboratory report came out, a pre-anesthetic checkup (PAC) was done from the anesthesia point of view. After getting fitness from anesthesia department, these patients were given a common date for the operation. After taking informed consent, pre-operative advised was given one day prior to surgery. On the day of operation, first patient was taken at 7:50 AM in operating room (OR-1) and anesthesia was given at 8:06, 8:01 and 8:08 A.M. on day 1, day 2 and day 3 respectively. After induction of anesthesia, the draping, the painting and the steps of LC were carried out by standard 4-port technique. The time when insufflation was started and gallbladder was extracted out was noted for all the cases. When the calots was identified, the process to take the next patient in operating room-2 was started and at the time of gallbladder extracted from the abdomen, anesthesia was given for the next case on adjacent OR. These sequential steps are depicted in Figure 1 (see PDF). There were 3 senior residents with experience of more than 3 years in surgical disciplines who assisted to one senior laparoscopic surgeon with 6 years of experiences in laparoscopic surgery. Two anesthetists were dedicated for each OR (Total 4 anesthetists as per rule 1-6). On the floor including scrub nurse, there was total 5 supporting staff for the help and for assisting in all cases. This sequential process was continuing up to 6 hours ± 40 minutes. All the patients were received standardized post-operative care for 24 hours and discharged on the next day. All the patients were followed up in outpatient department (OPD) at first and fourth week of discharge from the hospital. The data for 3 different days were collected on proforma and compared with the data for the routine practice for LC which was being done at our center. The mean for all the relevant results were analyzed and p-value < 0.05 was considered as statistically significant. Multivariate stepwise logistic regression was performed analyzing the various factors to decide the duration of surgery. Statistical analysis was performed using SPSS (12.0, Windows version; SPSS Inc., Chicago, IL, USA).

## Results:

In 3 different days, the total number of patients included in the study was 64 who were operated for laparoscopic cholecystectomy. On the first and second day 21 patients and on 3rd day, 22 patients were operated by following the rules 1 to 6. On all 3 days, the first case was taken in OR at 7:50 AM and intubated at 8:06 AM, 8:01 AM and 8:08 AM on first, second and third day respectively. The extubation of last case was done at 15:35 PM, 14:46 PM & 14:49 PM on first, second and third day respectively. The average duration of operation for each case was 28.85 minutes, 23.00 minutes and 21.50 minutes respectively on first, second and third day. The mean duration for each case was 24.45 minutes. The average duration for dissection of adhesions 2.0 +/- 0.5 minutes, elements of Calot's triangle 5.0 +/- 1.70 minutes, gallbladder releasing from its bed 3.0 +/- 1.8 minute, the abdominal cavity lavage and removal of gallbladder from the abdomen 3.8 +/- 0.8 minutes. When comparing the shortest and longest duration among all the 64 cases, it was 3rd case (on second day) that were operated very fast only in 9 minutes and 14th case (on first day) took maximum time that was 55 minutes. The intraoperative details of the present study are summarized in Table 1 (see PDF). The 98% of operated patient were female. The minimum and maximum age of the operated patients was 20 years and 62 years respectively. The mean age was 44.8 years. The maximum numbers of patient were in the range of 30 to 40 years of age. Drain was placed only in 15 patients. On comparing the time taken in cases without drain and with drain, it was statistically non-significant. In post-operative period 1 patient had severe pain abdomen, for which ultrasound was done which showed normal findings. Except one patient (who had severe pain abdomen) all patients were discharged on first post-operative day. At the first week of followed up, all patients were attended the clinic with histopathological report. All histopathological reports were consistent with features of chronic cholecystitis. Only 4 patients reported with mild pain at epigastric port. On evaluation there was nothing significant abnormality in these 4 patients. At four weeks of follow up, only 60% of patients attended the clinic with no complaint. Rest patients responded on phone and they all were doing well at home or the office. The intraoperative details of routine laparoscopic cholecystectomy over three different days are summarized in Table 2 (see PDF). Comparison of surgical outcomes between the 1-6 rule and routine practice, including operative numbers, duration, drain placement and complications, is shown in Table 3 (see PDF). The major difference between the two approaches was the significant time lost between cases in routine practice, ranging from 24 to 45 minutes, as detailed in Table 4 (see PDF). In contrast, the use of two operating rooms with four anaesthetists in the 1-6 rule approach minimized this wasted time.

## Discussion:

In ancient time, open cholecystectomy was the only option for gallstones diseases but for last two decades, laparoscopic cholecystectomy is considered as the gold standard treatment for gallstones diseases [7, 8- 9]. The first laparoscopic cholecystectomy was performed by Philippe Mouret in 1987 at Lyon, France [10, 11]. Initial learning curve for the laparoscopic cholecystectomy is higher and yields more complications as well as conversion into open [12]. But with the evolution and up gradation of laparoscopic machine it's not difficult to achieve a good learning curve within the 6 years of working in laparoscopic unit. Even in a good center, where huge number of laparoscopic cholecystectomy was being done, an average time of laparoscopic cholecystectomy was 31.9 ± 14.5 min [13] as compared to 24.4± 4 minute in present study. In one another observational study, it has been found that, an average time for lap cholecystectomy by an experienced surgeon was less than 1 hours and it may be more than 3 hours in case of difficulty like history of previous surgery and obesity [14]. In contrast to other study [13] where maximum time required was to dissect the gall bladder from the liver beds, here in our study this time was very minimal. This time could be saved by increased level of confidence and practice while taking the gall bladder away from liver as there is very less chance of any hazards during these steps except bleeding or spillage of bile. These two complications can be easily managed intraoperatively by coagulation with spatula and by proper lavage with water which takes very minimal time. In one study, an average time for LC for acute and chronic cholecystitis was 89 ± 21.2 and 57±33.4 minutes respectively as compared to only 24.3 minutes in present study which was statistically significant [15]. Another study found that an average time for LC in case of acute and chronic cholycystitis was 58 minutes and 45 minutes [13]. They also realized that with experience, careful dissection, patience and identification of vital anatomical structures, the LC can be safely completed even in difficult cases. The most important cause of conversion is the presence of dense and severe adhesions which was not found even in a single case in present study [7, 16]. In one study it was found that with maximum number of surgery with higher complication rate and more conversion into open, there is a strong correlation of experienced surgeon and minimal mean operative time [17]. An experienced surgeon can easily operate a complicated gallstone in a limited time frame with good outcome. One study showed that, usually the complicated cholecystitis was found in elderly with association of comorbid conditions [18]. In these complicated cases the operative time may be slightly longer as compared to no complicated cases. In previous studies, the most common comorbidities associated with gallstone disease were hypertension, ischemic heart disease, diabetes, and renal impairment, reflecting the metabolic and cardiovascular profile of these patients [19, 20]. In developing countries like India there is a long waiting list for the date of surgery for the gallstone diseases. There are multiple factors for this delay including higher incidence of gallstone diseases in India, limited health care center for laparoscopic surgery and provision to get limited operation room slot in government sector [3]. In government set up, even with all the best possible practice and support, the maximum number of laparoscopic surgeries done in 6 - 8 hours (Usual slot in government setting) was 6 to 8. The main reason for the minimum number of surgery done was the waste of time between two cases and more difficult cases because of late presentation and formation of giant gallstones [21]. In the present study by the innovative concept of rules 1-6, the maximum utilization of time especially the time between two cases was saved and an average 21.3 cases was operated in 6 hours of surgery.

## Limitations:

Smaller sample size limits us in generalizing the results. Need more number of laparoscopic hand instruments to maintain the sterility to implement this rule 1-6. We acknowledge that a multicenteric study with considering large number of sample size would have yielded robust results.

## Conclusion:

1 to 6 rules (1 consultant, 2 operation tables, 3 assistants, 4 anaesthetists, 5 nursing and supporting staff and 6 hours of surgery) may be the innovative idea as a policy decision for lap cholecystectomy for the maximum utilization of government infrastructures.

## Declarations:

## Ethics approval and consent to participate:

Need for approval was waived as this study was an observational study.

## Consent for publication:

Written informed consent was obtained from all the participants for publication. A copy of the written consent is available for review by the Editor-in-Chief of this journal on request.

## Availability of data and materials:

The data that support the findings of this study are available from the corresponding author, Dr Anil Kumar, upon reasonable request.

## Competing interests:

All authors have nothing to disclose.

## Funding:

All authors have no source of funding to disclose

## Author's Contribution:

Conception or design of the work: Dr Anil Kumar and Dr Ravi Shekhar

## Data collection:

Dr Shiv Kishor, Dr Abhyuday Kumar and Dr Shagufta Naaz

## Data analysis and interpretation:

Dr Anil Kumar and Dr Rekha Kumari

## Drafting the article:

Dr Anil Kumar and Dr Rekha Kumari and Dr Shiv Kishor

## Critical revision of the article:

Dr Ravi Shekhar and Dr Anil Kumar.

## Final approval of the version to be published:

All authors.

## Guarantor:

Dr Anil Kumar
